# Novel inhibitors of the calcineurin/NFATc hub - alternatives to CsA and FK506?

**DOI:** 10.1186/1478-811X-7-25

**Published:** 2009-10-27

**Authors:** Matthias Sieber, Ria Baumgrass

**Affiliations:** 1 Deutsches Rheuma-Forschungszentrum Berlin, Charitéplatz 1, D-10117 Berlin, Germany

## Abstract

The drugs cyclosporine A (CsA) and tacrolimus (FK506) revolutionized organ transplantation. Both compounds are still widely used in the clinic as well as for basic research, even though they have dramatic side effects and modulate other pathways than calcineurin-NFATc, too. To answer the major open question - whether the adverse side effects are secondary to the actions of the drugs on the calcineurin-NFATc pathway - alternative inhibitors were developed. Ideal inhibitors should discriminate between the inhibition of (i) calcineurin and peptidyl-prolyl *cis-trans *isomerases (PPIases; the matchmaker proteins of CsA and FK506), (ii) calcineurin and the other Ser/Thr protein phosphatases, and (iii) NFATc and other transcription factors. In this review we summarize the current knowledge about novel inhibitors, synthesized or identified in the last decades, and focus on their mode of action, specificity, and biological effects.

## Background

**Figure 1:**
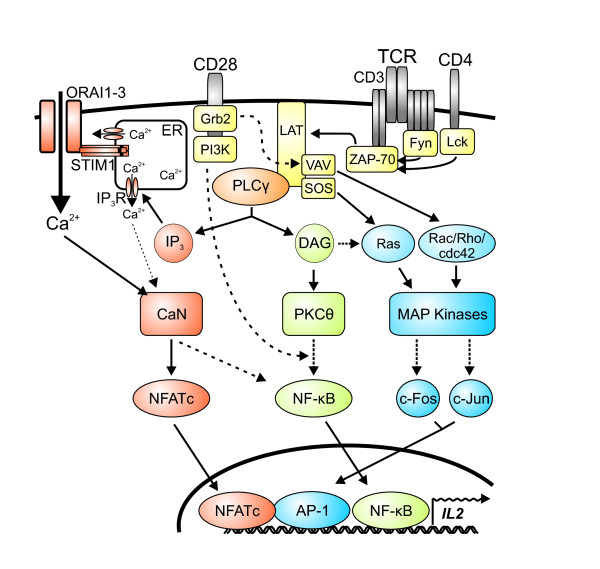
**Schematic overview about TCR-dependent signalling pathways**. Engagement of TCRs and costimulatory CD28 receptors promote signalling cascades of kinases and adaptor proteins (yellow). They trigger pathways resulting in the activation of the transcription factors NFATc (red), NF-κB (green) and AP-1 (blue). These transcription factors cooperate with each other during the activation of several genes, e.g. IL-2. Of special interest for this review is the calcineurin-NFATc pathway: IP_3_, generated by PLCγ (orange) via cleavage of PIP_2_, binds to the IP_3 _receptor (IP_3_R) and causes the release of Ca^2+ ^from the endoplasmatic reticulum (ER). This Ca^2+ ^depletion is sensed by STIM1, which is directly coupled to the ORAI CRAC channels. Influx of extracellular Ca^2+ ^into the cytosol activates calcineurin (CaN), leading to the dephosphorylation and nuclear translocation of NFATc. DAG: diacylglycerol; IP_3_: inositol-1,4,5-trisphosphate; PIP_2_: phosphatidylinositol-4,5-bisphosphate; PKCθ: protein kinase C theta; PLCγ: phospholipase C gamma.

The calcium-dependent serine/threonine (Ser/Thr) protein phosphatase calcineurin, discovered more than 30 years ago [1], is a key factor of a multitude of cell signalling processes, in particular in immune, neuronal and muscle cells. Targeting the phosphatase activity of calcineurin has revolutionized clinical transplantation. Calcineurin represents a hub of antigen specific T cell activation and differentiation (Figure 1). Inhibition of calcineurin totally blocks the adaptive immune response. Therefore, calcineurin is considered as "Achilles' heel of the immune system" [2].

### The Ser/Thr phosphatase calcineurin

Calcineurin, also named protein phosphatase 2B (PP2B), is a ubiquitously expressed cytosolic Ser/Thr protein phosphatase, highly conserved in eukaryotes. Calcineurin consists of two subunits - the enzymatic subunit A and the regulatory subunit B. The subunit A contains a calmodulin binding site and an autoinhibitory domain, which blocks the catalytic centre of the enzyme. Binding of Ca^2+ ^ions and calmodulin to calcineurin leads to a change of conformation and a subsequent unmasking of the active centre. Thereby, calcineurin activity is coupled to cytosolic calcium levels, which is a unique property of calcineurin among the Ser/Thr protein phosphatases [3]. Additionally, activity and localization of calcineurin is modulated by endogenous proteins, such as RCANs, Cabin1 or AKAP79. These regulatory proteins have been recently reviewed in detail [4,5].

### Calcineurin and NFATc

Calcineurin has the ability to dephosphorylate a broad range of proteins [6]. Some of the most important substrates are the transcription factors of the NFATc (nuclear factor of activated T cells) family: NFATc1 to NFATc4 [7]. NFATc regulates the expression of many genes by binding to DNA as dimers or in cooperation with other transcription factors. Among the regulated genes are cytokines such as IL-2, IL-4 and IFNγ or surface proteins such as CD40L and CD95L [8-10]. NFATc controls the expression of the endogenous calcineurin inhibitory protein RCAN1-4, thereby forming a negative feedback loop for the calcineurin-NFATc signalling [11].

Calcineurin seems to be the only protein phosphatase that dephosphorylates NFATc [12-14]. In resting T cells NFATc is highly phosphorylated and localized in the cytosol. Upon stimulation of T cells and subsequent calcium mobilization activated calcineurin dephosphorylates NFATc at 13 serine residues in the regulatory region [15], leading to its nuclear translocation by exposure of the nuclear localization sequences [16,17]. Concerted rephosphorylation of NFATc leads to its translocation into cytosol and abrogation of NFATc transcriptional activity [18,19].

NFATc is not only dephosphorylated by calcineurin but additionally interacts with calcineurin via two motifs binding at regions distinct from the catalytic centre of calcineurin. These motifs are named calcineurin binding region (CNBR)1 and CNBR2 or PxIxIT and LxVP according to their consensus sequences, respectively [20]. The PxIxIT region of NFATc binds even to inactive calcineurin and is responsible for basal NFATc-calcineurin interaction [21,22]. The LxVP motif interacts just with activated calcineurin, because its binding site at calcineurin is masked by the autoinhibitory domain [23,24]. Interaction of both NFATc motifs with calcineurin directs the regulatory region of NFATc into close vicinity to the active centre of calcineurin. This enables targeted dephosphorylation of specific NFATc serine residues by calcineurin. The PxIxIT calcineurin-binding motif of NFATc is shared by several other peptides and proteins binding to calcineurin. This motif may serve as a general calcineurin interface [25,26].

Calcineurin not only modulates the activity of NFATc but also several other transcription factors such as NF-κB, AP-1, and Elk1 [27-33]. In addition, calcineurin interferes with other signalling pathways such as TGF-β-dependent signalling and the MAPK cascade [33,34]. However, it is widely unknown, which components of these pathways are substrates or interaction partners of calcineurin and to which extent their dephosphorylation modulates the respective signalling.

In summary, calcineurin is unique in three aspects. First, it is the only Ca^2+^-dependent Ser/Thr protein phosphatase. Second, to date, only calcineurin is known to activate the NFATc transcription factors thereby controlling the expression of a broad range of genes. Third, the inhibition of calcineurin activity is so far the only effective therapeutic strategy to suppress the activation of memory CD4^+ ^and CD8^+ ^T cells [35,36]. The "classical" drugs targeting calcineurin activity and subsequently inhibiting NFATc activation are cyclosporin A (CsA) and FK506 (tacrolimus). Inhibitors of calcineurin are indispensable for treatment of transplantation patients and represent a valuable tool for basic research.

### CsA and FK506 - the classical calcineurin inhibitors

**Cyclosporin A **(CsA) and **FK506 **(tacrolimus) are widely used as effective immunosuppressants in the clinic, mainly in organ transplantation and dermatology [37-39]. Application of these compounds in basic research has substantially contributed to the elucidation of calcineurin-dependent signalling processes [40,41].

The immunosuppressive properties of CsA were discovered 1976 in animal models [42]. Since 1979 [43,44] CsA is indispensible for transplantation medicine. In 1987, FK506 was described as an alternative to CsA [45], followed by its first clinical application in 1989 [46]. Despite the widespread application of both compounds in the clinics their molecular mechanisms remained unclear until 1991. Then, Liu *et al*. identified calcineurin as the common target of both compounds if and only if they are complexed with the respective endogenous partners, the immunophilins. They showed that neither the isolated immunophilins nor the immunosuppressants alone but only immobilized immunophilin/immunosuppressant complexes are able to pull down the calcineurin/calmodulin protein from cellular extracts. These experiments clearly demonstrated that CsA and FK506 are not active calcineurin inhibitors by themselves but need binding to their endogenous matchmaker proteins to be activated in a gain of function mechanism [47,48]. Immunophilins, belonging to the class of peptidyl-prolyl *cis-trans *isomerases (PPIases), are involved in *de novo *protein folding and many other cellular functions [49,50]. Binding of CsA or FK506 to their respective major intracellular acceptor proteins cyclophilin A (CypA) and FK506 binding protein 12 (FKBP12) inhibits their PPIase activity. These CsA- and FK506-PPIase-complexes are noncompetitive inhibitors of calcineurin. Thereby, they severely limit the access of protein substrates to the active centre of calcineurin [51-54] and mask the docking site for the NFATc LxVP motif at calcineurin [24]. Thus, they inhibit the dephosphorylation of physiological targets of calcineurin. However, small molecular substrates like p-nitrophenyl phosphate (pNPP) are still being dephosphorylated [55,56]. The activity of other Ser/Thr protein phosphatases such as PP1, PP2A or PP2C is not affected by CsA- or FK506-complexes.

Although CsA and FK506 share a similar mode of action, they belong to different chemical classes. CsA is a fungal cyclic undecapeptide [57], whereas the bacterial FK506 belongs to the chemical class of macrolides [58,59].

Application of CsA and FK506 inhibits the T cell receptor (TCR)-dependent activation, proliferation, and differentiation of T cells. Both compounds inhibit the activation of NFATc and p65/NF-κB [60,61]. However, NF-κB regulated gene transcription is not completely blocked, due to additional, calcineurin-independent activation pathways for NF-κB [62]. Other cellular processes, such as CREB transcripitional activity [63] and proteasomal degradation of proteins [64,65], are modulated by CsA or FK506 treatment, too.

So far, CsA and FK506 are the only drugs suppressing not only the activation of naïve and effector T_H _cells, but in addition of memory T_H _cells. Therefore, the application of these drugs is crucial in particular for transplantation patients with high numbers of alloreactive memory/effector T cells, which cannot be controlled with calcineurin-inhibitor-free treatment protocols [35,66]. However, their use in clinical routine is often limited by severe side effects such as nephro- and neurotoxicity [67]. It is not known so far whether these side effects are mainly due to inhibition of calcineurin- or immunophilin-dependent mechanisms. Furthermore, it is not clear whether the modulation of the calcineurin-NFATc pathway or of other pathways and transcription factors cause the adverse side effects.

To dissect the different actions of CsA or FK506, alternative inhibitors should ideally discriminate not only between the inhibition of calcineurin and the other Ser/Thr protein phosphatases but in addition between the inhibition of calcineurin and PPIases as well as of NFATc and other substrates of calcineurin. Compounds having these properties would be more specific than CsA and FK506 and might cause fewer side effects in clinical applications. In basic research such compounds would help to identify and characterize different targets of calcineurin [68].

**Figure 2:**
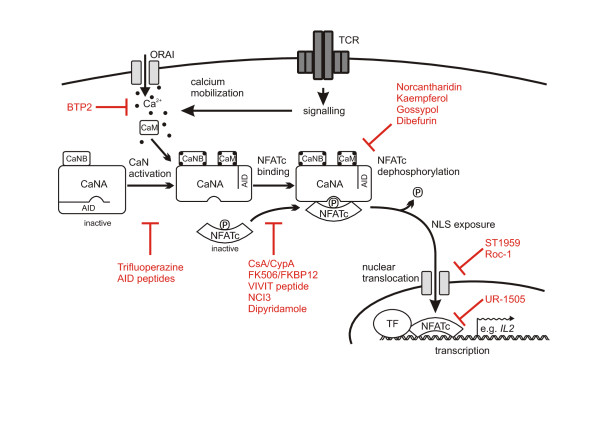
**Inhibition of the calcineurin-NFATc pathway at multiple levels**. Upon receptor induced Ca^2+ ^entry into the cell, calmodulin (CaM) and calcineurin B (CaNB) bind Ca^2+ ^ions and activate calcineurin by inducing a conformational switch of the subunit A (CaNA). Activated calcineurin binds NFATc via the PxIxIT and the LxVP motifs of NFATc and subsequently dephosphorylates the transcription factor. Dephosphorylated NFATc exposes its nuclear localization sequence (NLS) and is therefore shuttled into the nucleus, where it binds to the appropriate DNA sequences. NFATc exerts its transactivation effects often in combination with another transcription factor (TF). NFATc is deactivated by rephosphorylation and subsequent translocation into the cytosol. Different steps in this pathway are targeted by certain compounds to finally suppress NFATc-dependent gene expression. A selection of the most important and best characterized inhibitors of calcineurin-NFATc signalling is shown (in red) at their point of interference. AID: autoinhibitory domain of calcineurin A; TCR: T cell receptor.

Here we review several novel inhibitors of the calcineurin-NFATc pathway, which were discovered and partially characterized in the last decades. We summarize these inhibitors according to their mode of action (Figure 2), chemical structure, and inhibitory effects.

## Small molecular inhibitors

Binary complex formation with their respective PPIases is the prerequisite for the ability of CsA and FK506 to inhibit calcineurin. Thus, application of CsA and FK506 does not allow the discrimination between effects of these drugs on calcineurin and on the PPIases, respectively. Therefore, extensive research was undertaken to synthesize or identify derivatives of CsA and FK506 with mono-specificity for either inhibition of calcineurin or of PPIase activity (Table 1).

**Table 1:** Low molecular weight inhibitors of calcineurin-NFATc signalling

**Inhibitor**	**Mode of action**	IC_50_/K_d_*/K_i_**	**References**
*General protein phosphatase inhibitors inhibiting also calcineurin (CaN)*			

Endothall	binds to the active centre of PP1, PP2A, CaN	11,5 μM** ^a^	Tatlock JH 1997 [195]

FMPP	binds covalently to the active centre of PP1, PP2A, CaN	44 mM^b^	Born TL 1995 [88]

Norcantharidin	binds to the active centre of PP1, PP2A, CaN	31 μM^b^	Steward SG 2007 [90]

Okadaic acid	binds to the active centre of PP1, PP2A, CaN	4.6 μM^b^	Bialojan C 1988 [196]

Tyrphostins	bind possibly to the active centre of PP1, PP2A, CaN	21 - 62 μM^b^	Martin BL 1998 [89]

*Inhibitors of calcineurin-dependent signalling - CsA, FK506 and derivatives*			

CsA/CypA	complex blocks substrate access to the active centre of CaN	7 nM^a^	Fruman DA 1992 [197]

ISA247/CypA	complex blocks substrate access to the active centre of CaN	~100 nM^a^	Aspeslet L 2001 [75]

[Dat-Sar]^3^-CsA	blocks substrate access to CaN independent of CypA	1 μM^a^	Baumgrass R 2004 [73]

FK506/FKBP12	complex blocks substrate access to the active centre of CaN	0.4 nM^a^	Fruman DA 1992 [197]

Ascomycin/FKBP12	complex blocks substrate access to the active centre of CaN	0.7 nM^m^	Sinclair PJ 1996 [198]

Pimecrolimus/FKBP12	complex blocks substrate access to the active centre of CaN	0.4 nM^a^	Grassberger M 1999 [82]

*Inhibitors of calcineurin-dependent signalling - alternative inhibitory compounds*			

1,5-dibenzoyloxymethyl-norcantharidin	binds to the active centre of CaN	7 μM^a^	Baba Y 2003 [101]

AM404	inhibits NFATc-DNA binding	10 μM^c^	Caballero FJ 2007 [132]

BTP1	inhibits of NFATc dephosphorylation in cells	60 nM^d^	Trevillyan JM 2001 [118]

BTP2	decreases Ca^2+ ^influx into cytosol	10 nM^c^	Ishikawa J 2003 [119]

Dibefurin	inhibits enzymatic activity of CaN	44 μM^e^	Brill GM 1996 [110]

Dipyridamole	disrupts CaN-NFATc binding	~10 μM^a^	Mulero C 2009 [111]

Gossypol	inhibits enzymatic activity of CaN	17 μM^a^	Baumgrass R 2001 [199]

INCA1		0.5 μM* ^f^	Roehrl MH 2004 [114]
		
INCA2	disturb CaN-NFATc complex formation by covalent binding to CaN	0.12 μM* ^f^	Roehrl MH 2004 [114]
		
INCA6		0.8 μM* ^f^	Roehrl MH 2004 [114]

Kaempferol	inhibits enzymatic activity of CaN	51.3 μM^a^	Wang H 2008 [94]

Lie120	inhibits enzymatic activity of CaN	5 μM^a^	Klettner A 2001 [80]

NCI3	disturbs CaN-NFATc binding in cells	2 μM^c^	Sieber M 2007 [113]

PD 144795	inhibits enzymatic activity of CaN	~3 μM^a^	Gualberto A 1998 [109]

Roc-1	enhances rephosphorylation and nuclear export of NFATc	5~25 nM^c^	Proksch P 2005 [136]

ST1959/DL111-IT	enhances nuclear export of NFATc	13.5 μM	Lindstedt R 2009 [128]

Thiopental	inhibits CaM-dependent activity of CaN	0.18 mM^a^	Humar M 2004 [97]

Trifluoperazine	inhibits binding of CaM to CaN	8 μM^g^	Aussel C 1995 [144]

Tropisetron	inhibits CaN-dependent NFATc transactivation	~50 μM^c^	Vega Lde L 2005 [138]

UR-1505	inhibits NFATc binding to DNA	100~300 μM^h^	Román J 2007 [133]

WIN 53071	alters NFATc binding to DNA	3.4 μM^h^	Baine Y 1995 [141]

*Inhibitors of calcineurin-dependent signalling - unknown mode of action*			

Caffeic Acid Phenethyl Ester	N.D.	0,1~1 μM^c^	Marquez N 2003 [146]

KRM-III	N.D.	5 μM^c^	Jung EJ 2009 [145]

NFAT-68	N.D.	0.63 μM^c^	Burres NS 1995 [152]

YM-53792	N.D.	50 nM^c^	Kuromitsu S 1997 [149]

Punicalagin	N.D.	< 5 μM^g^	Lee SI 2008 [153]

Imperatorin	N.D.	37 μM^c^	Marquez N 2004 [154]

Quinolone alkaloid compounds 1 and 3	N.D.	3.4 μM^c^	Jin HZ 2004 [155]

Impressic acid	N.D.	12.6 μM^c^	Cai XF 2004 [159]

oleanane triterpenoid compound 3	N.D.	4.6 μM^c^	Dat NT 2004 [162]

Gomisin N	N.D.	1.33 μM^c^	Lee IS 2003 [163]

N.D.: not determined

Indicated methods for IC_50_/K_d_/K_i _determination: a - phosphatase assay on R_II _phosphopeptide; b - phosphatase assay on pNPP; c - NFAT dependent reporter gene expression; d - IL-2 promoter dependent reporter gene expression; e - phosphatase assay on 4-methylumbelliferyl phosphate; f - displacement of VIVIT peptide; g - IL-2 secretion assay; h -EMSA for NFATc; i - interference with CaN-NFATc binding assay; k - binding assay to CaN; l - phosphatase assay on NFATc; m - cell proliferation assay

### CsA derivatives

CsA is a cyclic undecapeptide causing CypA and calcineurin inhibition via different parts of the molecule. CsA residues 2-9 are responsible for CypA binding, while CsA residues 4-7 are involved in calcineurin binding.

Modifications in position 3 [69], position 6 [70,71] or position 8 [72] resulted in some CsA derivatives, such as **[(*S*)α-methylthiosarcosine^3^]-CsA**, **[N-Methyl-Ala^6^]-CsA **and ** [D-Diaminopropyl^8^]-CsA**, which bind to CypA but are not able to inhibit calcineurin's activity, neither in their uncomplexed form nor in the complex with CypA.

Several other CsA derivatives substituted in position 3, e.g. **[(*R*)α-Methylsarcosine^3^]CsA **and ** [Dimethylaminoethylthiosarcosine^3^]CsA **have been found to inhibit calcineurin without prior formation of a complex with cyclophilin 18 or inhibition of its isomerase activity. However, both compounds show affinity towards CypA and can therefore not be considered monospecific [73].

Modifications of CsA in the position 1 change the affinity of the derivatives towards CypA. The derivative **[MeBm_2_t]^1^-CsA **has a lower binding affinity than CsA [74], but the binary complex retains the immunosuppressive capacity. The derivative **ISA247 **(voclosporin) has a higher affinity to CypA than CsA [75,76] and has the potential to be administered in lower concentrations. Therefore it might be less toxic than CsA and is under investigation in phase II and III trials for psoriasis patients [77,78].

### FK506 derivatives

FK506 has several derivatives with the same mode of action. Among them are the immunosuppressive compounds **FK520 **(ascomycin) and **pimecrolimus**. Other FK506 derivatives are monospecific for FKBP12 binding and inhibition, such as **L-685,818 **(FK506BD) [79] and **V-10,367 **[80]. However, so far there are no FK506 derivatives with monospecific action on calcineurin.

Several of the immunosuppressive derivatives have been characterized in detail.

**FK520 **[81] is a naturally occurring FK506 derivative containing an ethyl group in the position 21 and is used as an immunosuppressant *in vitro *and in rodent models. Several semisynthetic immunosuppressive compounds are derived from FK520. **Pimecrolimus **(SDZ AM 981, 33-epi-chloro-33-desoxy-ascomycin) [82], is routinely used in the topical treatment of inflammatory skin diseases. It is more lipophilic than FK506 and therefore more affine to the skin, has low systemic effects, and does not induce skin atrophy, in contrast to topical steroids. The derivative **L-732,531 **(32-O-(1-hydroxyethylindol-5-yl)-ascomycin) binds poorly to FKBP12, but the stability of the L-732,531-FKBP12-calcineurin-complex is much higher compared to the complex with FK506 [83]. This might be the reason that L-732,531 has less severe side effects in a murine transplant model [84].

Interestingly, FK506 and its related, naturally occurring immunosuppressive compound **rapamycin **(sirolimus) share the FKBP-binding domain but differ in their effector domains [85]. Rapamycin/FKBP12 does not bind to calcineurin [47], but exerts its immunosuppressive and antiproliferative effects via inhibition of the mTOR/Akt signalling. This signalling node is part of costimulatory and IL-2 receptor signalling pathways [86]. Rapamycin is often used in clinical routine in prolonged treatment of transplantation patients after initial CsA or FK506 application. Rapamycin suppresses posttransplantational malignant neoplasia due to its antiproliferative properties [87].

### Inhibitors acting on the calcineurin molecule

The catalytic centre of calcineurin (PP2B) shares the structure and conformation with other Ser/Thr protein phosphatases, namely PP1 and PP2A. Therefore, several inhibitors targeting the active centre of calcineurin additionally inhibit these protein phosphatases, too. Among them are **4-(fluoromethyl)phenyl phosphate (FMPP) **[88], **tyrphostins **[89] and **norcantharidin **[90]. However, some inhibitors, such as **okadaic acid **or **endothall**, show different affinities towards different phosphatases. By using selected concentration ranges of these inhibitors it is possible to inhibit mainly PP1 and PP2A, but calcineurin to a lower degree. Okadaic acid inhibits PP1 and PP2A in nanomolar, but calcineurin in micromolar concentrations [91]. **Pyrethroid insecticides **do not possess any ability to inhibit calcineurin [92,93], contrary to an earlier report.

The following compounds (Table 1; see additional file 1 for IUPAC names or chemical structures) inhibit calcineurin specifically among the Ser/Thr protein phosphatases. They might act mainly as noncompetitive inhibitors, such as CsA and FK506, but in contrast to them they do not need a matchmaker protein.

**Kaempferol**, a natural flavonol, inhibits the phosphatase activity of purified calcineurin against pNPP and R_II _phosphopeptide. Kaempferol binds to the catalytic domain of calcineurin A and acts independently of any matchmaker protein. Protein phosphatase 1 and alkaline phosphatase activity are not inhibited by this compound. Kaempferol suppresses IL-2 gene expression in Jurkat T cells [94,95]. Surprisingly, it also inhibits the calcineurin-independent TNFα-induced NF-κB activation in HEK293 cells [96].

**Barbiturates **such as thiopental, pentobarbital, thiamylal and secobarbital are inhibitors of the phosphatase activity of calcineurin [97]. They inhibit the calmodulin-mediated dephosphorylation of the R_II _phosphopeptide [55] in enzymatic assays, as well as NFATc dephosphorylation, NFATc nuclear translocation and cytokine production in T cells. These effects do not depend on binding of the barbiturates to their GABA receptor and its subsequent signalling. Additionally, barbiturates inhibit AP-1 activation independently of calcineurin [98]. Thiopental suppresses NF-κB activation via unknown mechanisms [99]. In lymphocytes, thiopental decreases antigen- but not mitogen-induced proliferation, IL-2 expression and IFNγ expression [100].

**1,5-dibenzoyloxymethyl-norcantharidin **is a derivative of norcantharidine [101,102]. Among other compounds, it was synthesized according to a computational interaction model of norcantharidin carboxylate with the catalytic centre of calcineurin. It has been selected by screening for specific binding to calcineurin, but not to PP1 and PP2A. 1,5-dibenzoyloxymethyl-norcantharidin inhibits the dephosphorylation of pNPP and the R_II _phosphopeptide by calcineurin. However, no data about inhibition of protein dephosphorylation in cell-free assays or in cells have been reported for this compound.

**Gossypol**, a cell permeable polyphenole identified in cotton plants, inhibits noncompetitively the enzymatic activity of calcineurin but of none of the other Ser/Thr protein phosphatases. The inhibition of pNPP and R_II _phosphopeptide dephosphorylation is reversible and is independent of immunophilins or other matchmaker proteins. Gossypol inhibits the nuclear translocation of NFATc in activated T cells. The reported effect of gossypol on calcineurin-calmodulin interaction [103] cannot account for these specific effects, as gossypol only partially prevents the binding of calmodulin to calcineurin and just at very low concentrations of calmodulin. Gossypol shows immunosuppressive effects in mice [104] and inhibitory effects on growth, cell signalling and development in *Dictyostelium *cells. These effects are specific for calcineurin as they can be diminished by calcineurin overexpression [105]. However, gossypol has been reported to additionally inactivate other enzymes than calcineurin such as dehydrogenases [106], phospholipase A2 [107] and topoisomerase II [108].

**Lie120**, a thiazole derivative, inhibits the enzymatic activity of calcineurin but not the PPIase activity of FKBPs or cyclophilins. Dephosphorylation of pNPP and R_II _phosphopeptide is inhibited by Lie120 in cell-free assays. In neuronal cell lines it prevents the FK506-sensitive H_2_O_2_-triggered activation of JNK. However, Lie 120 is toxic at concentrations higher than 5 μM [80].

**PD 144795**, a benzothiophene derivative, blocks the enzymatic activity of calcineurin in cell lysates. In Jurkat T cells transfected with an HIV-1 LTR fragment, PD 144795 inhibits the calcineurin-dependent binding of p53 and NF-κB to the HIV promoter element. These inhibitory effects are abolished by overexpression of calcineurin [109].

**Dibefurin**, a fungal phenolic compound, inhibits the enzymatic activity of calcineurin against the small molecular substrate 4-methylumbelliferyl phosphate. Furthermore, it shows suppressive effects in both assays, mixed lymphocyte reaction (MLR) and lymphocyte cytotoxicity analysis [110].

Compounds inhibiting the enzymatic activity of calcineurin are supposed to block the dephosphorylation of all protein substrates. Therefore, only compounds targeting specific calcineurin-substrate interactions but not the general phosphatase activity of calcineurin might be able to dissect the action of calcineurin on different substrates. Recently, different efforts were made to identify such compounds, interfering specifically with calcineurin-NFATc interactions in T cells.

**Dipyridamole**, a drug clinically used for stroke treatment, is suggested to affect the interaction of NFATc with calcineurin, because it competes with fluorescence-labelled RCAN1-CIC peptide (see below) for binding to calcineurin. Dipyridamole does not interfere with the phosphatase activity of calcineurin on R_II _phosphopeptide in cell-free assays. It suppresses ionomycin-induced NFATc2 nuclear translocation in Jurkat T and U-2 osteosarcoma cell lines, and blocks subsequently NFATc-dependent reporter gene and cytokine expression [111]. Dipyridamole inhibits TNFα production in activated PBMC [112].

**NCI3**, a pyrazolopyrimidine derivative, does not influence the enzymatic activity of calcineurin in cell-free systems. However, NCI3 inhibits NFATc dephosphorylation and nuclear translocation, IL-2 secretion and cell proliferation upon stimulation of Jurkat or primary human T cells. NFATc-dependent reporter gene expression is more sensitive to NCI3 than NF-κB, whereas AP-1-dependent transcription is not influenced. These effects are diminished by calcineurin overexpression. An effect of NCI3 on the calcineurin-substrate interface is postulated as it partially displaces the VIVIT peptide, an oligopeptide derived from the PxIxIT-calcineurin binding motif of NFATc (see below) [113].

**INCA **compounds are a group of chemically unrelated substances selected in a screening for inhibition of NFATc-calcineurin interaction. INCA-1, 2 and 6 bind covalently but reversibly to calcineurin at the residue Cys266. Subsequently, steric changes mask the binding site for NFATc and VIVIT peptide. INCA-2, but not INCA-6, inhibits the enzymatic activity of calcineurin. INCA-6 inhibits the dephosphorylation of NFATc and its nuclear import in ionomycin-stimulated Cl.7W2 murine T cell line and, consequently, the expression of IFNγ and TNFα. However, general cytotoxicity has been reported for all INCA compounds, ruling out their use in primary cells [114,115].

### Inhibitors not acting directly on the calcineurin molecule

Inhibitors of calcineurin-NFATc signalling may not only act on calcineurin itself, but also up- or downstream of the calcineurin-NFATc interaction or dephosphorylation processes. Among the possibilities are effects of compounds on calcium mobilization, on the nuclear translocation of NFATc or on NFATc-DNA-binding (Figure 2). Inhibitors acting downstream of calcineurin activation might be more specific to suppress just NFATc activation than CsA or FK506 complexes.

**BTPs **or 3,5-Bis(trifluoromethyl)pyrazoles are a batch of NFATc modulators with different modes of action [116,117].

**BTP1 and BTP3 **(compounds 1 and 2 in Djuric *et al*.) are supposed to interfere with calcineurin-dependent NFATc activation, because calcineurin activity against R_II _phosphopeptide and phosphorylated Elk1 is not inhibited in enzyme assays and in cell lysates. BTP1 and BTP3 diminish activation-dependent NFATc dephosphorylation, its nuclear translocation in primary T cells and cell lines as well as subsequent cytokine production and cell proliferation. It is assumed that NF-κB or AP-1 activation are not affected by BTP1 and BTP3 [118].

**BTP2 **(compound 3 in Djuric *et al*., alternative name YM-58483) dose-dependently enhances TRPM4, a Ca^2+^-activated nonselective cation channel. Thereby, BTP2 decreases CRAC-channel-dependent Ca^2+ ^influx due to depolarization of lymphocyte cell membranes. Subsequently, the activation of calcineurin is diminished, leading to a reduced NFATc-driven promoter activity and IL-2 production in Jurkat T cells. AP-1-driven promoter activity is not influenced. BTP2 also inhibits the proliferation and Ca^2+^-dependent cytokine production in stimulated human CD4^+ ^T cells [119-121] and the expression of IL-4 and IL-5 in an antigen-stimulated murine T_H_2 T cell clone. *In vivo *studies show an inhibition of antigen-induced airway-inflammation [122], of donor anti-host cytotoxic T lymphocyte activity and IFNγ production in graft versus host disease, and of delayed-type hypersensitivity response in mice [123]. Inhibition of Ca^2+^-dependent functional responses of human neutrophils and granulocyte-differentiated HL60 cell line was also observed [124]. However, it is unclear to which extent these observed effects are caused by inhibition of calcineurin, because other Ca^2+^-dependent processes are suppressed, too. In particular, the activation of the calmodulin-dependent kinases (CaMK) plays an important role in T cell activation and inflammatory responses.

**BTP A-285222 **(compound 19 in Djuric *et al*.) has immunosuppressive effects in an animal model but exhibits severe side effects, such as neurotoxicity. The molecular mode of action of BTP A-285222 is not known, nevertheless many effects on different cell types are observed. It was found that cytokine production of T cells is reduced by 80% in BTP A-285222-treated mice [125], that agonist-induced NFATc3-dependent IL-6 production is inhibited in myometrial arteries and that proliferation of isolated vascular smooth muscle cells is impaired [126,127].

**ST1959**, a 3,5-diaryl-s-triazole derivative which is also named DL111-IT/contragestazol, inhibits T cell activation, proliferation and cytokine production by enhancing the nuclear export of NFATc2. NFATc2 de- and rephosphorylation are not influenced. NF-κB- and AP-1-dependent gene transcription are reported to be not affected [128]. The compound shows immunosuppressive effects in several animal models of autoimmune diseases, such as colitis and host versus graft disease [129,130].

**AM404**, a product of the acetaminophen (paracetamol) catabolism [131], inhibits NFATc2-DNA binding and transcriptional activity in Jurkat T cells, but not in cell lysates. It is postulated that AM404 inhibits the nuclear translocation of dephosphorylated NFATc. AM404 does not inhibit Ca^2+ ^influx, disturbs only slightly the dephosphorylation of NFATc2 in cells and does presumably not interfere with the signalling pathways leading to NF-κB or AP-1 activation. However, AM404 suppresses IL-2 and TNFα transcription, T cell proliferation and cytokine release in Jurkat T cells after αCD3/28 stimulation [132].

**UR-1505 **is a pentafluoropropoxy derivative of salicylic acid [133]. It blocks the binding of NFATc to DNA upon ionomycin stimulation but has no effect on the nuclear translocation of NFATc. Therefore, UR-1505 effects are not due to NFATc-export inhibition or enhanced re-phosphorylation. The activation of NF-κB and AP-1 seems to be not affected. UR-1505 inhibits αCD3/CD28-induced, but not JAK/STAT-induced T cell proliferation and IL-5 as well as IFNγ expression. UR-1505 shows anti-inflammatory properties in rat colitis models [134]. **Triflusal**, another salicylic acid derivative, inhibits not only NFATc-DNA complex formation, but additionally NF-κB activation [135].

**Rocaglamide derivatives **Roc-1, 2 and 3 inhibit the activation-induced NFATc1 translocation into the nucleus. It is supposed that elevated kinase activities of p38 MAPK and JNK by Roc-1, 2 and 3 cause an increased NFATc re-phosphorylation. This inactivation of NFATc leads to a reduced expression of IL-2, IL-4, IFNγ and TNFα. The nuclear localization of c-Jun, a potential subunit of AP-1, is also inhibited. Surprisingly, only NFATc- but not AP1- or NF-κB-dependent reporter gene transcription is suppressed by the inhibitors in the selected concentration range (up to 100 nM) [136].

**Tropisetron**, an antagonist of the serotonin receptor, inhibits NFATc-dependent signalling caused by overexpression of the constitutively active calcineurin construct ΔCaM-AI [137]. Therefore, a target at or downstream of calcineurin activity was suggested. Tropisetron inhibits the transcriptional activities of NFATc, NF-κB and AP-1 in PMA/ionomycin-, but not TNFα-stimulated Jurkat T cells. Tropisetron suppresses the phosphorylation of p38 MAPK and JNK but not the phosphorylation of ERK 1 and 2. It inhibits IL-2 production in primary T cells upon SEB stimulation [138]. Tropisetron ameliorates acetic-acid-induced colitis in rats [139].

*Venkatesh et al*. selected 14 compounds in a screening of a library with 16,000 substances for inhibitors of GFP-NFATc3 nuclear translocation in HeLa cells. Most of them interfered with calcium mobilization and therefore calcineurin activation [140].

**WIN 53071 **alters NFATc-DNA complex formation in intact cells but not in cell-free binding assays. It does not inhibit the enzymatic activity of calcineurin against R_II _phosphopeptide. WIN 53071 inhibits Ca^2+^-dependent IL-2 expression in primary human lymphocytes, MLR and NFATc-driven reporter gene expression [141].

**Trifluoperazine **binds to calmodulin and inhibits its interaction with calcineurin [142]. Therefore, trifluoperazine inhibits calcineurin activation and suppresses the dephosphorylation of R_II _phosphopeptide in cell lysates [143] and IL-2 expression of αCD3/PMA-activated Jurkat T cells. Due to its mode of action, also other calmodulin-dependent but calcineurin-independent processes are modulated, such as phosphatidylserine synthesis [144].

### Inhibitors with unknown mode of action

Several compounds, belonging to different chemical classes, were found to inhibit NFATc-dependent gene expression and other NFATc-mediated cellular effects. However, the underlying molecular mechanisms of the compounds listed in this chapter were not elucidated in detail by the respective authors.

**KRM-III **inhibits NFATc-, but not NF-κB-dependent reporter gene expression in PMA/ionomycin stimulated Jurkat T cells. KRM-III diminishes the proliferation of TCR-stimulated murine T cells and MLR. Upon oral application to mice, KRM-III reduces the IL-2 levels in blood after αCD3-injection, and diminishes delayed type hypersensitivity responses and MOG-induced experimental autoimmune encephalomyelitis [145].

**Caffeic Acid Phenethyl Ester (CAPE)**, a phenolic compound derived from honey bee propolis, inhibits IL-2 promoter activity and cytokine synthesis, NF-κB binding to DNA in PMA-stimulated Jurkat cells (but not IκBα degradation), NFATc dephosphorylation after PMA/ionomycin stimulation, and the DNA binding of a pGal4-NFATc2(1-415) fusion protein in cells. CAPE inhibits not only the percentage of cells expressing the activation markers CD25, CD69, and ICAM-1 at the cell surface but also the relative intensity of fluorescence in the positive cell population [146]. It is a potent inhibitor of antigen- and mitogen-induced T-cell proliferation, cytokine production [147], and NF-κB activation [148]. The precise mode of action of CAPE remains unclear.

**YM-53792 **suppresses NFATc-, but not AP-1- and NF-κB-driven promoter activities and the formation of NFATc-DNA complexes in stimulated Jurkat cells. YM-53792 inhibits IL-2 gene promoter activity and the expression of IL-2, IL-4 and IL-5 in stimulated human peripheral blood mononuclear cells. It was assumed that YM-53792 specifically inhibits the calcineurin-NFATc pathway, but the molecular mechanism is not elucidated [149].

**Quinazolinediones **and **pyrrolopyrimidinediones**, selected by screening of a compound library, inhibit NFATc-dependent reporter gene transcription in Jurkat cells [150]. No further examination of their mode of action has been reported. Recent data suggest that the inhibitory effect of quinazolinediones such as WIN 61058 [141] is independent of calcineurin but due to inhibition of the monocarboxylate transporter MCT1, which is crucial in the export of catabolic lactate from activated blastic T cells [151].

**NFAT-68 **and **NFAT-133 **are no proteins but in fact fungal aromatic compounds. They do not interfere with calcineurin phosphatase activity against 4-methylumbelliferyl phosphate, but inhibit NFATc-driven reporter gene transcription, MLR and lymphocyte toxicity [152].

Many traditional medical plants, parts of plants or specific preparations from them often have anti-inflammatory properties. To identify the responsible natural ingredients and to search for novel calcineurin-NFATc-pathway inhibitors, different libraries were recently screened for inhibitors of NFATc-dependent reporter gene expression. Unfortunately, most of the selected compounds were not further characterized concerning their mode of action and their effects on the inhibition of other transcription factors and pathways.

**Punicalagin**, isolated from the fruit of *Punica granatum*, inhibits NFATc nuclear translocation and DNA binding. It diminishes αCD3/28-induced IL-2 production of CD4^+ ^T cells and shows slight suppression of MLR. Immunosuppressive effects of punicalagin were observed in a PMA-induced edema mouse model [153].

**Imperatorin **isolated from *Oppopanax chironium*(L.), a furanocumarin, inhibits both NFATc transcriptional and DNA-binding activities. It blocks the expression of the reporter gene luciferase controlled by NFATc- or IL-2-dependent promoter region, but not the expression under control of an NF-κB- or AP-1-dependent promoter. Imperatorin suppresses the proliferation of SEB-stimulated T cells [154].

**Quinolone alkaloids **from the *Evodia rutaecarpa *fruit inhibit NFATc- and NF-κB-dependent reporter gene expression in Jurkat T cells. Several of these alkaloids suppress NFATc signalling stronger than NF-κB without affecting the viability of the Jurkat T cell line [155].

Several compounds, extracted from of Asian plants, inhibit NFATc-dependent reporter gene expression: **phenolic constituents 2 and 3 **of *Desmos chinensis *[156], **gymnasterkoreayne G**, a polyacetylene isolated from the leaves of *Gymnaster koraiensis *[157], **compound 1 **from *Ribes fasciculatum *var. *chinense *[158], **Impressic acid **[159], **lignans **[160], and **diterpenoids **[161], contained in *Acanthopanax koreanum *root, **oleanane triterpenoid compound 3**, contained in the fruits of *Liquidambar formosana*, [162], as well as **gomisin N **and **schisandrol A**, lignans from *Schisandra chinensis *[163].

## Inhibitory peptides and proteins from pathogens

Protein domains from interaction partners of calcineurin are usually the point of origin to design inhibitory peptides. These specific peptides, targeting the binding of calcineurin to selected partners and substrates, might be more specific than CsA- or FK506-complexes (Table 2). They should help to delineate the interaction mechanisms of calcineurin interaction with other signalling partners. Additionally, modifications of these peptides such as fragment shortening and point mutations will support these efforts. However, often these peptides have low cell membrane permeability, even when they are synthesized with specific modifications such as leading peptide sequences containing oligo-arginines.

**Table 2:** Peptides and proteins inhibiting calcineurin-NFATc signalling

**Inhibitor peptide**	**Mode of action**	IC_50_/K_d_*/K_i_**	**References**
CaN_457-482_-AID		10 μM^a^	Perrino BA 1999 [167]
	mask the active centre of CaN		
CaN_424-521_-AID		2.5 μM^a^	Sagoo JK 1996 [164]

mNFATc2_106-121 _- SPRIEIT		12 μM^i^	Aramburu J 1998 [21]
	block CaN-NFATc interaction		
VIVIT peptide		0.5 μM* ^k^ < 1 μM^l^	Roehrl MH 2004 [114] Aramburu J 1999 [171]

AKAP79_330-357_	blocks CaN-NFATc interaction	1.5 μM^a^	Dell'Acqua ML 2002 [173]

RCAN1		60 nM* ^k^	Chan B 2005 [177]
		
RCAN1-4_141-197 _- exon7	block CaN-NFATc interaction	70 nM* ^k^	Chan B 2005 [177]
		
RCAN1-4_143-163 _- CIC peptide		1.25 μM* ^k^	Mulero MC 2009 [111]

LxVPc1 peptide	blocks CaN-NFATc interaction and modulates enzymatic activity of CaN	~3 μM^a^	Rodriguez A 2009 [24]

RCAN1-4_95-118 _- SP repeat peptide	masks the active centre of CaN	91.5 μM** ^a^	Vega RB 2002 [185]

VacA	inhibits translocation of NFATc	N.D.	Gebert B 2003 [187]

A238L		N.D.	Miskin JE 1998 [190]
	block CaN-NFATc interaction		
A238L_200-213_		0.6 μM^k^	Miskin JE 2000 [194]

N.D.: not determined

Indicated methods for IC_50_/K_d_/K_i _determination: a - phosphatase assay on R_II _phosphopeptide; b - phosphatase assay on pNPP; c - NFAT dependent reporter gene expression; d - IL-2 promoter dependent reporter gene expression; e - phosphatase assay on 4-methylumbelliferyl phosphate; f - displacement of VIVIT peptide; g - IL-2 secretion assay; h - EMSA for NFATc; i - interference with CaN-NFATc binding assay; k - binding assay to CaN; l - phosphatase assay on NFATc; m - cell proliferation assay

### Inhibitory peptides

**AID fragments**, derived from the autoinhibitory domain of the calcineurin A subunit were the first examined inhibitory peptides for calcineurin. These peptides, containing the residues 424-521 (**AID**_**424-521**_), are potent inhibitors of the phosphatase activity by blocking the access of protein substrates to the catalytic centre of calcineurin [164]. A peptide spanning the residues 457-482 (**AID**_**457-482**_) of calcineurin represents the core inhibitory motif [165,166]. This peptide is already able to suppress the dephosphorylation of the R_II _phosphopeptide in phosphatase assays. However, additional autoinhibitory elements are present within the calcineurin region 420-457. Therefore, the peptides containing the extended AID region AID_420-511 _and AID_328-511 _were three- to fourfold more potent to inhibit R_II _phosphopeptide dephosphorylation compared to the AID_457-482 _peptide [167]. The 11R-AID_457-482 _peptide, containing eleven arginine residues, is reported to be indeed cell-permeable for selected cell types. It inhibits apoptosis of excitatory neurons [168] and caerulein-induced zymogen activation in pancreatic acinar cells [169].

**PxIxIT peptides **are derived from the conserved calcineurin-docking motif PxIxIT found in NFATc and other proteins [170]. Peptides or protein fragments containing the PxIxIT element compete with NFATc for binding to calcineurin and impair thereby the binding and dephosphorylation of NFATc1, c2 and c4 in cell-free enzyme assays. In cells overexpressing PxIxIT peptides, the phosphatase activity of calcineurin and therefore the dephosphorylation of other substrates are not impaired [21-23,25].

The **VIVIT 16 mer oligopeptide**, designed by selective amino acid exchange, possesses 25 times higher efficiency to inhibit NFATc dephosphorylation compared to the original NFATc2 16 mer **SPRIEIT peptide **[171]. Overexpression of GFP-VIVIT fusion protein in Jurkat T cells inhibits NFATc- but not NF-κB-dependent reporter gene expression. Therefore, the VIVIT peptide is more selective than CsA and FK506 complexes which inhibit the activation of both transcription factors. 11R-VIVIT peptide is claimed to be cell-permeable in selected cell types [172], but there are contrary experimental experiences.

A peptide derived from the calcineurin-anchoring protein AKAP79 containing the PIAIIIT motif (**AKAP79**_**330-357**_) binds to purified calcineurin. In contrast to other PxIxIT peptides, this motif inhibits the phosphatase activity against the R_II _phosphopeptide. Overexpression of AKAP79_330-357 _in HEK293 cells antagonizes the interaction between AKAP79 and calcineurin [173].

The endogenous inhibitory protein CABIN1 contains a conserved PEITVT motif. A peptide spanning the residues **2078-2115 of rat CABIN1 **binds to calcineurin [174], and human **CABIN1**_**2143-2220 **_overexpression in Jurkat T cells inhibits NFATc dephosphorylation and NFAT-dependent luciferase expression [175]. Both fragments overlap at the KFPPEITVTPP sequence. Therefore, this motif is assumed to participate in the CABIN1-calcineurin interaction.

RCAN1, an endogenous modulator of calcineurin activity (also named DSCR1 or calcipressin1), is expressed in several splice variants which differ in their N termini but share an identical C terminus. In this review, the amino acid designation has been adapted from the splice variant RCAN1-4 [176], although several cited publications use the designation of the splice variant 1-1. **RCAN1-exon7 **(RCAN1-4 residues 141-197), containing the C-terminus, binds to full-length calcineurin and to a catalytic core fragment of calcineurin as well as full-length RCAN1. Both, RCAN1-exon7 and full-length RCAN1, inhibit competitively the dephosphorylation of pNPP in enzyme assays and the calcineurin-mediated nuclear translocation of NFATc3 in the BHK cell line after their overexpression [177]. Initially, the PKIIQT motif in this region (RCAN1-4_181-186_) was considered to mimic the NFATc PxIxIT motif and to inhibit NFATc-calcineurin interaction, but a peptide spanning the residues 178-191 did not compete with VIVIT peptide for binding to calcineurin. Recent experiments revealed that the **RCAN1-4**_**136-163 **_fragment contains a region named calcineurin-inhibitor calcipressin 1 (CIC) motif, which is displaced from calcineurin by the VIVIT peptide [178]. The **RCAN1-4**_**143-163 **_CIC fragment binds to calcineurin A with high affinity, competes with the binding of VIVIT peptide, and inhibits NFATc2 nuclear translocation as well as NFATc-dependent reporter gene expression in transfected COS-7 cells. Importantly, this fragment does not interfere with the phosphatase activity of calcineurin towards R_II _phosphopeptide and homologous regions are found in the related proteins RCAN2 (residues 147-167) and RCAN3 (residues 183-203) [111]. This fragment contains the "true" PxIxIT motif of RCANs - PSVVVH, which binds to the same hydrophobic pocket of calcineurin as the VIVIT peptide. Therefore, the PSVVVH peptide (**RCAN1-4**_**149-166**_) competes with the regulatory region of NFATc2 or GST-CABIN1 for binding to calcineurin [179].

A peptide derived from the C-terminus of the yeast RCAN homologue Rcn2 (**Rcn2**_**252-265**_), containing the PSITVN motif, is able to compete with the VIVIT peptide for binding to human calcineurin, too. Consequently, deletion of this motif in Rcn2 abolishes its inhibition of calcineurin signalling in yeast [180].

The **African Swine Fever Virus protein A238L **contains a similar motif the PKIIITG motif (see below).

**LxVP peptides **are derived from the conserved calcineurin-docking NFATc motif LxVP and compete with NFATc for the binding to activated calcineurin. The NFATc isoforms differ in the affinity of their LxVP motifs towards calcineurin [181].

**Pep3 **is derived from the CNBR2 of NFATc3 (residues 321-406 of murine NFATc3). This 16-amino acid oligopeptide (mNFATc3_385-400_) contains the LxVP motif, binds to purified and cellular calcineurin and competes with GST-CNBR2 for binding to calcineurin. Retroviral overexpression of Flag-Pep3 in the murine D10G4.1 T_H_2 cell line impaired the expression of IL-5, IL-6 and IL-13 and the nuclear translocation of NFATc3 after PMA/calcium ionophore stimulation. NFATc3, but not NFATc2 and NF-κB activation is affected by Pep3 [182].

The **LxVPc1 peptide**, spanning the 15 amino acids of human NFATc1 371-385, disrupts calcineurin-NFATc1 and c2 binding. GST-LxVPc1 binds to calcineurin more efficiently than any of the PxIxIT motifs of NFATc1 to c4. The GST-LxVPc2 fusion peptide from NFATc2 was unable to bind to calcineurin under the same conditions. The LxVPc1 peptide inhibits calcineurin phosphatase activity on the R_II _phosphopeptide and increases the phosphatase activity on pNPP. Overexpression of GFP-LxVPc1 fusion protein in HeLa cells inhibits NFATc2 dephosphorylation and nuclear translocation upon ionophore treatment; in Jurkat T cells it inhibits NFATc2 dephosphorylation and the expression of luciferase under control of the IL-2 or RCAN1-4 promoter upon PMA/ionophore stimulation [23,24].

**Protein fragments based on other motifs **were derived from CABIN1 and RCAN1. The protein fragment **CABIN1**_**700-901 **_inhibits the dephosphorylation of the R_II _phosphopeptide by calcineurin in a noncompetitive manner. Overexpression of CABIN1_700-901 _in HEK293 cells coexpressing constitutively active calcineurin inhibits the dephosphorylation of NFATc2, its nuclear translocation and luciferase reporter gene expression under NFAT control. Overexpression of this fragment in Jurkat T cells suppresses the expression of luciferase controlled by the IL-2 promoter upon PMA/ionomycin stimulation [183].

RCAN1 and 2 contain a SP repeat motif binding to the catalytic centre of calcineurin. The SP repeat peptide (**RCAN1-4**_**95-118**_), which can be phosphorylated by MAPK and GSK-3, simulates a substrate for calcineurin and thereby inhibits calcineurin activity against R_II _phosphopeptide in a competitive manner in cell-free assays. This inhibitory effect is independent of the phosphorylation status of the peptide. However, overexpressed RCAN1 fragments containing only the SP repeat domain do not suppress calcineurin-NFATc signalling in cells [184,185]. A peptide containing the CIC motif and the C-terminal 30 amino acids of RCAN1 blocks dephosphorylation of the R_II _phosphopeptide by calcineurin, but neither the CIC containing peptide nor the C-terminus alone. It is suggested, that the C-terminal inhibitory motifs have to be in close proximity to calcineurin via binding of the CIC motif [179].

### Pathogen proteins

Calcineurin represents a crucial hub of T cell receptor-dependent signalling and controls the T cell activation mainly via NFATc dephosphorylation. Targeting this mechanism would enable pathogens to evade the host immune responses. Therefore, several viruses and bacteria have developed proteins inhibiting calcineurin-NFATc-dependent signalling. Characterizing these proteins might help to understand host defence mechanisms.

**VacA **is a protein from *H. pylori*, which inhibits the nuclear translocation of NFATc. In addition, VacA blocks ionomycin-induced increase of intracellular Ca^2+ ^level, and the activation of the MKK3/6-p38 MAPK pathway. These data suggest multiple modes of VacA action, not all of them seem to be calcineurin-NFATc-dependent [186]. However, VacA inhibits T cell activation, proliferation and IL-2 secretion in Jurkat cell lines and primary human CD4^+ ^T cells [187,188]. VacA is imported into the T cell via the receptors CD18 and LFA-1 [189]. The expression of these cell surface proteins varies in different cell types, resulting in a different magnitude of inhibitory effects.

**A238L**, a protein of the african swine fever virus, seems to have different functions: first, to bind to calcineurin and inhibit its phosphatase activity and thus calcineurin-dependent pathways [190]; second, to suppress the acetylation and transcriptional activation of the transcription factors NFATc2, NF-κB, and c-Jun by inhibition of transactivation of the transcriptional co-activator CREB binding protein/p300 by PKCθ in stimulated human T cells [191]; and third, to inhibit the activation of JNK [192]. Overexpression of A238L reduces calcineurin phosphatase activity against R_II _phosphopeptide in cell lysates and diminishes NFATc-dependent reporter gene expression in transfected porcine RS-2 kidney cells [190]. It is speculated that A238L only inhibits the dephosphorylation of such NFATc residues which might be crucial for its transactivation function but has no effect on the dephosphorylation of the other residues required for nuclear translocation or DNA binding [193]. Effects of A238L on NFATc-dependent gene transcription are abolished by co-overexpression of the constitutively active calcineurin construct ΔCaM-AI or NFATc2 in Jurkat T cells [193]. Interestingly, A238L binds also to CypA, but this interaction seems to have no effect on A238L-calcineurin interaction [190].

The fragment **A238L**_**157-238 **_contains a PxIxIT site (here: PKIIITG) and binds to calcineurin with high affinity. The 14 mer oligopeptide derived from this fragment **A238L**_**200-213 **_binds to calcineurin even with a faster rate than the SPRIEIT peptide of porcine NFATc1 [194].

## Commentary

### Clinical application

CsA and FK506 as well as some of their derivatives have become indispensible drugs to prevent transplant rejection and to treat dermatologic and autoimmune disorders. However, these drugs have a narrow therapeutic window. To reduce their adverse side effects different approaches were tested and some of them are still under investigation.

One strategy is to reduce the concentration of CsA and FK506, respectively, by combined application together with other immunosuppressive drugs having different modes of action, such as mycophenolate, rapamycin or monoclonal antibodies (e.g. Alemtuzumab). Another approach, especially in atopic dermatitis is to diminish systemic effects of the drugs by topical application of derivatives with enhanced lipophilic properties, such as pimecrolimus. A third approach is to apply CsA in low doses, thus taking advantage not only of its immunosuppressive, but also of its immunomodulatory properties [39].

Recently, some drugs originally introduced for applications other than inflammation turned out to act as inhibitors of calcineurin/NFATc activation. Among them are barbiturates, tropisetron and the acetaminophen/paracetamol catabolite AM404.

### Application in basic research

Small molecular inhibitors and inhibitory peptides are valuable tools for a functional "knock-down" of cellular components to study the impact of different proteins, processes and pathways on specific cellular functions. Ideal inhibitors have to be monospecific, cell permeable and non-toxic. Most of the novel inhibitors, however, are not as well characterized as the "classical" inhibitors CsA and FK506.

Here we recommend the application of several inhibitors in basic research based on the intended investigations (Figure 2).

#### Inhibition of different Ser/Thr phosphatase activities

Norcantharidin inhibits PP1, PP2A and calcineurin with comparable efficacy. Okadaic acid or calyculin A can be used to discriminate between calcineurin and the other main Ser/Thr protein phosphatases when applied in the nanomolar concentration range. Under this condition they inhibit PP1 and PP2A, which account for more than 90% of the cellular Ser/Thr phosphatase activity, and additionally PP4, PP5 and PP6, but they fail to inhibit calcineurin.

#### Inhibition of calcineurin phosphatase activity without modulation of PPIases activities

Gossypol and kaempferol are useful to inhibit the phosphatase activity of calcineurin not only in enzymatic assays but also in primary cells due to their low cytotoxicity. They inhibit calcineurin without the need for a matchmaker protein. Both compounds, however, additionally inhibit several other cellular enzymes. Inhibitors of calcium signalling, e.g. BTP2 and trifluoperazine, suppress the activation of calcineurin but they also act on other Ca^2+^- or calmodulin-dependent processes, such as Ca^2+^-dependent PKCs or CaMKI/II. Peptides derived from the auto-inhibitory domain of calcineurin inhibit the phosphatase activity with high specificity, but their application is limited due to their reduced cell permeability.

#### Inhibition of NFATc dephosphorylation

NCI3 and dipyridamole are cell permeable compounds which inhibit NFATc dephosphorylation in cells without inhibition of the phosphatase activity of calcineurin in enzymatic assays. INCA-6 inhibits NFATc dephosphorylation and can be used in cell-free systems but is not recommended in primary cells due to its cytotoxicity. VIVIT peptide competes with NFATc for binding to calcineurin and is appropriate to inhibit NFATc dephosphorylation in cell-free assays.

#### Inhibition of NFATc-dependent gene transcription

BTP1, ST1959 and Roc-1 inhibit NFATc-dependent gene transcription presumably downstream of the calcineurin-NFATc interaction. These compounds are supposed to have no or low inhibitory effects on NF-κB or AP-1 activation.

Several other novel inhibitors of NFATc-dependent gene transcription have been isolated or synthesized. However, most of them have not been characterized so far and their molecular mode of action remains to be elucidated. Therefore, these compounds cannot be recommended as tools to dissect and define mechanisms of calcineurin action in the complex signalling network of cells.

## Conclusion

In summary, CsA and FK506 are firmly established in the clinical routine. Several approaches are applied or under investigation to limit their side effects. In basic research, several more specific, although less well characterized, inhibitors of the calcineurin-NFATc axis can be utilized as alternatives. So far, their widespread application is hindered by a limited commercial availability.

## List of abbreviations used

AID: autoinhibitory domain of calcineurin; AP-1: activator protein 1; CsA: cyclosporin A; CypA: cyclophilin A; FKBP: FK506-binding protein; CaN: calcineurin; CaM: calmodulin; GFP: green fluorescent protein; MAPK: mitogen activated protein kinase; MLR: mixed lymphocyte reaction; NFATc: nuclear factor of activated T cells, cytosolic; NF-κB: nuclear factor κB; PMA: phorbol-12-myristate-13-acetate; pNPP: para-nitrophenyl phosphate; PP: protein phosphatase; PPIase: peptidyl-prolyl *cis-trans *isomerase; TCR: T cell receptor; TGF-β: transforming growth factor β

## Competing interests

The authors declare that they have no competing interests.

## Authors' contributions

Both authors contributed to the writing of this manuscript and approved the final manuscript.

## Supplementary Material

Additional file 1 **IUPAC names or chemical structures of low molecular weight inhibitors of CaN-NFATc signalling**. The table summarizes the IUPAC names or structural formulas of the low molecular weight inhibitors of CaN-NFATc signalling reviewed in this article. Click here for file
