# Supplementary material for: Novel inhibitors of the calcineurin/NFATc hub - alternatives to CsA and FK506?
Source: Cell Commun Signal. 2009 Oct 27;7:25. doi: 10.1186/1478-811X-7-25 (PMC2774854; doi:10.1186/1478-811X-7-25)
Supplement: Additional file 1 — IUPAC names or chemical structures of low molecular weight inhibitors of CaN-NFATc signalling. The table summarizes the IUPAC names or structural formulas of the low molecular weight inhibitors of CaN-NFATc signalling reviewed in this article. [file 1478-811X-7-25-S1.DOC]

**IUPAC names or chemical structures of low molecular weight inhibitors of CaN-NFATc signalling**

| **Inhibitor, trivial name** | **IUPAC name or chemical structure** | **Reference** |
| --- | --- | --- |
| 1,5-dibenzoyloxymethyl-norcantharidin | 1,5-Dibenzoyloxymethyl-7-oxa­bicyclo[2.2.1]hep­tane-2,3-dicarboxylic anhydride | Baba Y 2003 [101] |
| AM404 | (5*Z*,8*Z*,11*Z*,14*Z*)-*N*-(4-hydroxyphenyl)icosa-5,8,11,14-tetraenamide | Caballero FJ 2007 [132] |
| BTP1 | 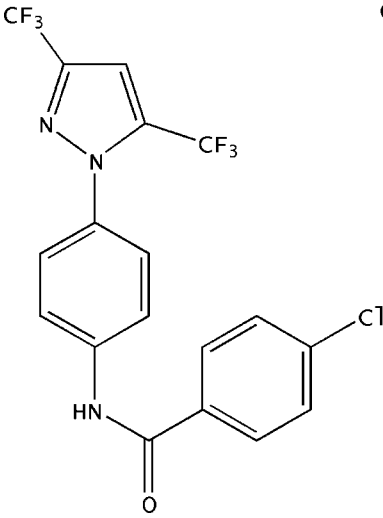 | Trevillyan JM 2001 [118] |
| BTP2 | 5-bis(trifluoromethyl)pyrazol-1-yl]phenyl]-4-methylthiadiazole-5-carboxamide | Ishikawa J 2003 [119] |
| BTP3 |  | Trevillyan JM 2001 [118] |
| BTP A-28522 | 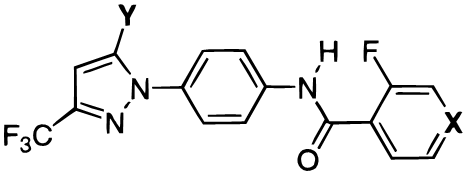 X=N Y=OCHF_2_ | Djuric SW 2000 [116] |
| Caffeic Acid Phenethyl Ester | phenethyl (E)-3-(3,4-dihydroxyphenyl)prop-2-enoate | Marquez N 2004 [146] |
| Dibefurin | 4,5,6-trihydroxy-7-methyl-(1*H*,3*H*)-dihydroisobenzofuran dimer | Brill GM 1996 [110] |
| Dipyridamole | 2,2',2'',2'''-(4,8-di(piperidin-1-yl)pyrimido[5,4-*d*]pyrimidine-2,6-diyl)bis(azanetriyl)tetraethanol | Mulero C 2009 [111] |
| Gossypol | 1,1',6,6',7,7'-hexahydroxy-3,3'-dimethyl-5,5'-diisopropyl[2,2'-binaphthalene]-8,8'-dicarboxaldehyde | Baumgrass R 2001 [199] |
| INCA1 | 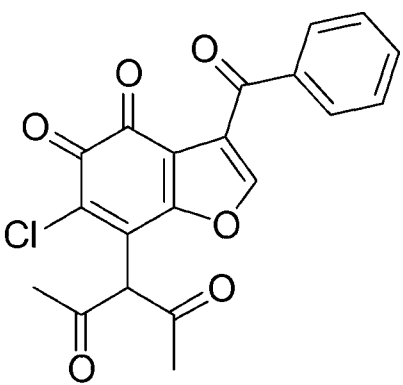 | Roehrl MH 2004 [114] |
| INCA2 | 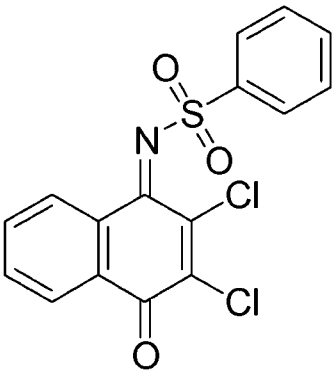 | Roehrl MH 2004 [114] |
| INCA6 | 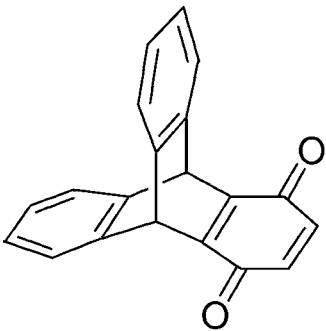 | Roehrl MH 2004 [114] |
| Kaempferol | 3,4',5,7-tetrahydroxyflavone | Wang H 2008 [94] |
| Lie120 | substituted Thiazol | Klettner A 2001 [80] |
| NCI3 |  | Sieber M 2007 [113] |
| NFAT-133 | 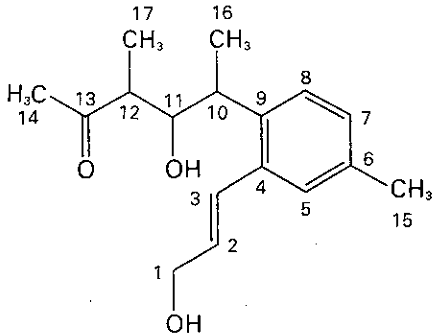 | Burres NS 1995 [152] |
| PD 144795 | Benzothiophene-2-carboxamide, 5-methoxy-3-(1-methylethoxy)-1-oxide | Gualberto A 1998 [109] |
| Roc-1 | rocaglamide, (1R,2R,3S,3aR,8bS)-1,8b-dihydroxy-6,8-dimethoxy-3a-(4-methoxyphenyl)-N,N-dimethyl-3-phenyl-2,3-dihydro-1H-cyclopenta[b][1]benzofuran-2-carboxamide | Proksch P 2005 [136] |
| Roc-2 | 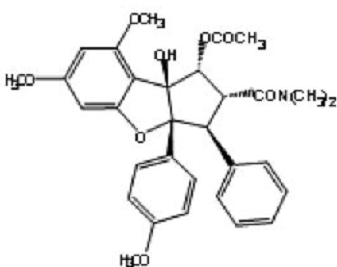  acetylrocaglamide | Proksch P 2005 [136] |
| Roc-3 | 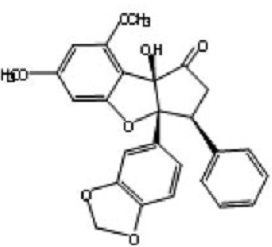  1-oxo-11,12-methylendioxyrocaglaol | Proksch P 2005 [136] |
| ST1959/DL111-IT | 5-(2-Ethylphenyl)-3-(3-methoxyphenyl)-1H-1,2,4-triazole | Lindstedt R 2009 [128] |
| Thiopental | 5-Ethyl-5-pentan-2-yl-2-sulfanylidene-1,3-diazinane-4,6-dione | Humar M 2004 [97] |
| Trifluoperazine | 10-[3-(4-methylpiperazin-1-yl)propyl]-2-(trifluoromethyl)phenothiazine | Aussel C 1995 [144] |
| Triflusal | 2-acetyloxy-4-(trifluoromethyl)benzoic acid | Aceves M 2004 [135] |
| Tropisetron | [(1R,5S)-8-Methyl-8-azabicyclo[3.2.1]octan-3-yl] 1H-indole-3-carboxylate | Vega Lde L 2005 [138] |
| UR-1505 | 2-Hydroxy-4-(2,2,3,3,3-pentafluoropropoxy)benzoic acid | Román J 2007 [133] |
| WIN 53071 |  | Baine Y 1995 [141] |
| KRM-III | 1, 4-aryl-2-mercaptoimidazole | Jung EJ 2009 [145] |
| YM-53792 | 3-(13-Hydroxytridecyl)-1-[13-(3-pyridyl)tridecyl]pyridiniumchloride | Kuromitsu S 1997 [149] |
| NFAT-68 | 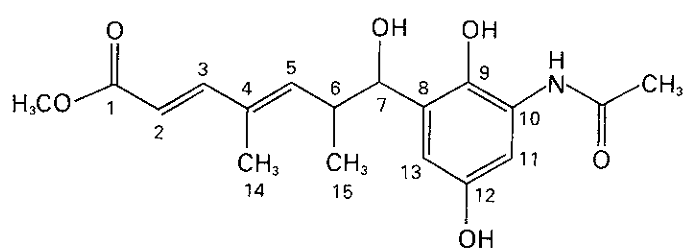. | Burres NS 1995 [152] |
| Punicalagin | 2,3-(S)-Hexahydroxydiphenoyl-4,6-(S,S)-gallagyl-D-glucose | Lee SI 2008 [153] |
| *Desmos chinensis* compound 2 | negletein, 5,6-dihydroxy-7-methoxy-2-phenylchromen-4-one | Kiem PV 2005 [156] |
| *Desmos chinensis* compound 3 | 2',3'-dihydroxy-4',6'-dimethoxydihydrochalcone | Kiem PV 2005 [156] |
| Gymnasterkoreayne G | *erythro*-8(S)-9(Z),16-heptadecadi-ene-4,6-diyne-2,3,8-triol | Dat NT 2005 [157] |
| *Ribes fasciculatum* compound 1 | *threo*-(7S,8R)-1-(4-hydroxyphenyl)-2-[4-(E)-propenylphenoxy]-propan-1-ol | Dat NT 2005 [158] |
| Imperatorin | 9-(3-Methylbut-2-enoxy)furo[3,2-g]chromen-7-one | Marquez N 2004 [154] |
| Quinolone alkaloid compound 1 | 2-Nonyl-4(1*H*)-quinolone | Jin HZ 2004 [155] |
| Quinolone alkaloid compound 3 | 2-Undecyl-4(1*H*)-quinolone | Jin HZ 2004 [155] |
| Impressic acid | 3α, 11α-Dihydroxylup-20(29)-en-28-oic acid | Cai XF 2004 [159] |
| oleanane triterpenoid compound 3 | 3a-acetoxy-25-hydroxy-olean-12-en-28-oic acid | Dat NT 2004 [162] |
| Gomisin N | 1,2,3,12-tetramethoxy-6,7-dimethyl-10,11-methylenedioxy-5,6,7,8-tetra­hydro­dibenzo­[*a*,*c*]cyclooctene, | Lee IS 2003 [163] |
| Schisandrol A | 5,6,7,8-tetrahydro-1,2,3,10,11,12-hexamethoxy-6,7-dimethyl-dibenzo(a,c)cycloocten-6-ol | Lee IS 2003 [163] |

IUPAC names and chemical structures were obtained from the indicated references or from the PubChem compound database (http://pubchem.ncbi.nlm.nih.gov)
